# β1, 4-*N*-acetylgalactosaminyltransferase III modulates cancer stemness through EGFR signaling pathway in colon cancer cells

**DOI:** 10.18632/oncotarget.1981

**Published:** 2014-05-18

**Authors:** Mei-Ieng Che, John Huang, Ji-Shiang Hung, Yo-Chuen Lin, Miao-Juei Huang, Hong-Shiee Lai, Wen-Ming Hsu, Jin-Tung Liang, Min-Chuan Huang

**Affiliations:** ^1^ Graduate Institute of Anatomy and Cell Biology, National Taiwan University College of Medicine, Taipei, Taiwan; ^2^ Department of Surgery, National Taiwan University Hospital, Taipei, Taiwan; ^3^ Departments of Medical Research, National Taiwan University Hospital, Taipei, Taiwan; ^4^ Research Center for Developmental Biology and Regenerative Medicine, National Taiwan University, Taipei, Taiwan

**Keywords:** B4GALNT3, EGFR, colorectal cancer, LacdiNAc, cancer stem cells

## Abstract

Cancer stem cells are cancer cells characterized with tumor initiating capacity. β1,4-N-acetylgalactosaminyltransferase III (B4GALNT3) synthesizes GalNAcβ1-4GlcNAc (LacdiNAc) which contributes to self-renewal of mouse embryonic stem cells. We previously showed that B4GALNT3 overexpression enhances colon cancer cell malignant phenotypes *in vitro* and *in vivo*. However, the role of B4GALNT3 in cancer stemness remains unclear. We found that B4GALNT3 expression was positively correlated with advanced stages and poor survival in colorectal cancer patients. Knockdown of B4GALNT3 using small interfering (si) RNAs in colon cancer cell lines (HCT116, SW480, HCT15, and HT29 cells) decreased sphere formation and the expression of stem cell markers, *OCT4* and *NANOG*. The expression of *B4GALNT3* was upregulated in colonospheres. Interestingly, we found that B4GALNT3 primarily modified *N*-glycans of EGFR with LacdiNAc by *Wisteria floribunda* agglutinin (WFA) pull down assays. B4GALNT3 knockdown suppressed EGF-induced phosphorylation of EGFR and its downstream signaling molecules. Furthermore, EGF-induced degradation of EGFR was facilitated. In addition, EGF-induced migration and invasion were significantly suppressed by B4GALNT3 knockdown. Taken together, these data suggest B4GALNT3 regulates cancer stemness and the invasive properties of colon cancer cells through modifying EGFR glycosylation and signaling. Our results provide novel insights into the role of LacdiNAc in colorectal cancer development.

## INTRODUCTION

Colorectal cancer is one of the most common malignancy worldwide [1]. Over 1.2 million new colorectal cancer cases and 600,000 colorectal cancer-related deaths are reported every year. Surgery remains the main treatment for colorectal cancer to provide long term survival. After surgery, chemotherapy and radiotherapy are also applied for relapse prevention. However, the 5-year survival rate of stage III patients is 30-60%, and recurrence is the primary course of treatment failure [2]. Recently, increasing evidence suggests that the recurrence of colorectal cancer is mediated by cancer stem cells inside the tumor [3-5].

In the cancer stem cell model, the tumor cell mass contains a subpopulation of cells which possesses the ability to self-renew and differentiate into different cancer cells within the tumor [6]. In addition to tumor initiation and progression, cancer stem cells are also considered to be responsible to cancer invasion, recurrence, and drug resistance. Therefore, identifying factors that can regulate cancer stem cell properties is important for improving the treatment of colorectal cancer.

Dysregulated glycosylation is commonly found in various cancers, including colorectal cancer [7]. Altered glycans are often associated with tumor progression and have been demonstrated to regulate cell proliferation, migration, invasion, adhesion, angiogenesis, and evasion from immune system. Several tumor-associated carbohydrates have been found in tumors, such as sialyl Lewis X, sialyl Lewis A, Tn, sialyl-Tn, and T. Interestingly, glucosylceramide synthase, an enzyme catalyzes ceramide glycosylation, was recently found to maintain the properties of breast cancer stem cells [8]. However, roles of glycosyltranferases in cancer stem cells remain largely unknown.

*β1,4-N-acetylgalactosaminyltransferase III* (*B4GALNT3*) is mainly expressed in stomach, colon, and testis [9]. B4GALNT3 transfers N-acetylgalactosamine (GalNAc) to non-reducing N-acetylglucosamine (GlcNAc) to form GalNAcβ1-4GlcNAc (LacdiNAc) in an *in vitro* enzymatic assay [10]. B4GALNT3 has been reported to be highly expressed in epithelial cells of gastric mucosa and is suggested to be responsible for the formation of LacdiNAc [11]. In our previous study, we found that B4GALNT3 overexpression enhances malignant phenotypes of colon cancer cells *in vitro*, and increases tumor growth and metastasis *in vivo* [12]. However, the underlying mechanisms are still unclear. A recent study showed increased LacdiNAc expression enhances self-renewal of mouse embryonic stem cells and B4GALNT3 knockdown decreases the expression of LacdiNAc [13]. We therefore hypothesized that B4GALNT3 could enhance the cancer stem-like cell property in colorectal cancer. In this study, we found that B4GALNT3 is upregulated in advanced stages colorectal cancer and associated with poor prognosis. B4GALNT3 knockdown suppresses EGF-induced sphere formation, migration and invasion of colon cancer cells. The mRNA level of *B4GALNT3* is increased in colonospheres. Notably, B4GALNT3 can modify the LacdiNAc structure on EGFR. B4GALNT3 knockdown inhibits EGFR activity and downstream signaling as well as facilitates EGFR degradation. These findings demonstrate that B4GALNT3 can regulate cancer stemness and the invasive properties through modifying EGFR glycosylation and activity. Our findings not only provide novel insights into the significant role of LacdiNAc in colorectal cancer stemness and but also suggest B4GALNT3 as a potential therapeutic target.

## RESULTS

### B4GALNT3 Expression in Primary Colorectal Tumors

**Figure 1:**
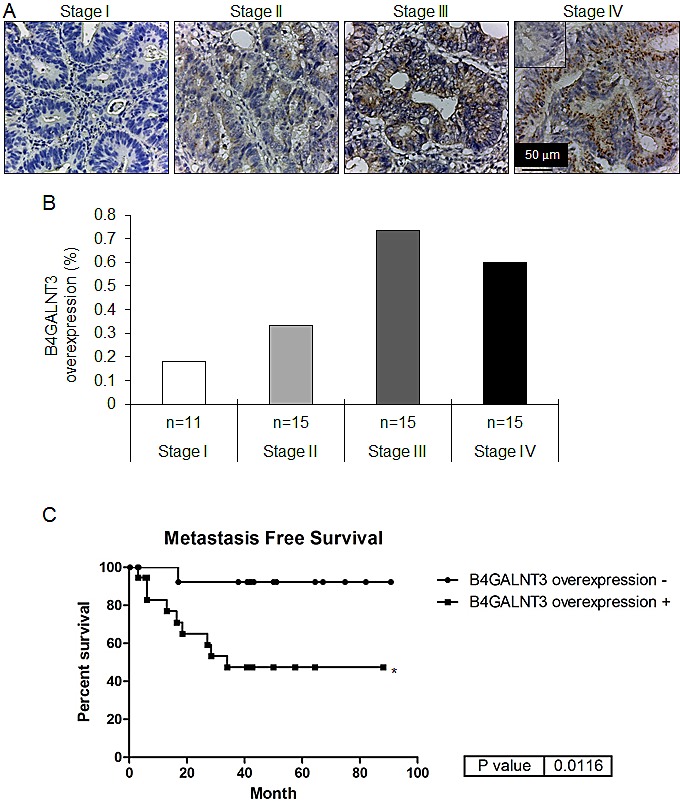
Immunohistochemistry of B4GALNT3 in human colorectal cancer (A) B4GALNT3 expression in different stage of colorectal tumors. The inset in the Stage IV tumor indicates negative staining by the control rabbit IgG. The scale bar is 50 μm. Magnification: × 400. (B) B4GALNT3 overexpression in colorectal tumors (n = 56) is positively correlated with AJCC stage. The B4GALNT3 expression was analyzed by immunohistochemical stain. n indicates the patient number in each stage group. (C) Kaplan-Meier survival curves for patients with colorectal cancer. Correlation between B4GALNT3 overexpression and metastasis free survival in patients was analyzed. *P < 0.05.

To investigate the expression pattern of B4GALNT3 in colorectal tumors, immunohistochemistry was performed. Representative images of immunohistochemistry showed B4GALNT3 expression in different stage of colorectal tumors compared with their surrounding non-tumorous tissues (Figure 1A). Statistical results from immunohistochemistry of different stage of colorectal cancers showed that B4GALNT3 overexpression was observed in 18.18% (2/11) of stage I colorectal cancer and in 33.33% (5/15) of stage II colonrectal cancer. There was a higher percentage of B4GALNT3 overexpression in 73.33% (11/15) of stage III colorectal cancer and in 60.00% (9/15) stage IV colorectal cancer. Chi-square test showed that B4GALNT3 overexpression in colorectal tumors is positively associated with advanced American Joint Committee on Cancer stages (p = 0.01918; Figure 1B) by immunohistochemical stain. Further investigation on survival data with these patients (n= 56) revealed that high expression of B4GALNT3 correlated with higher metastatic (p= 0.0116; Figure 1C). Our results indicate B4GALNT3 as a marker of poor prognosis of colorectal cancer and suggest a metastasis-promoting function of the glycosyltransferase in colorectal cancer.

### B4GALNT3 regulates stem-like potential in colon cancer cells

**Figure 2:**
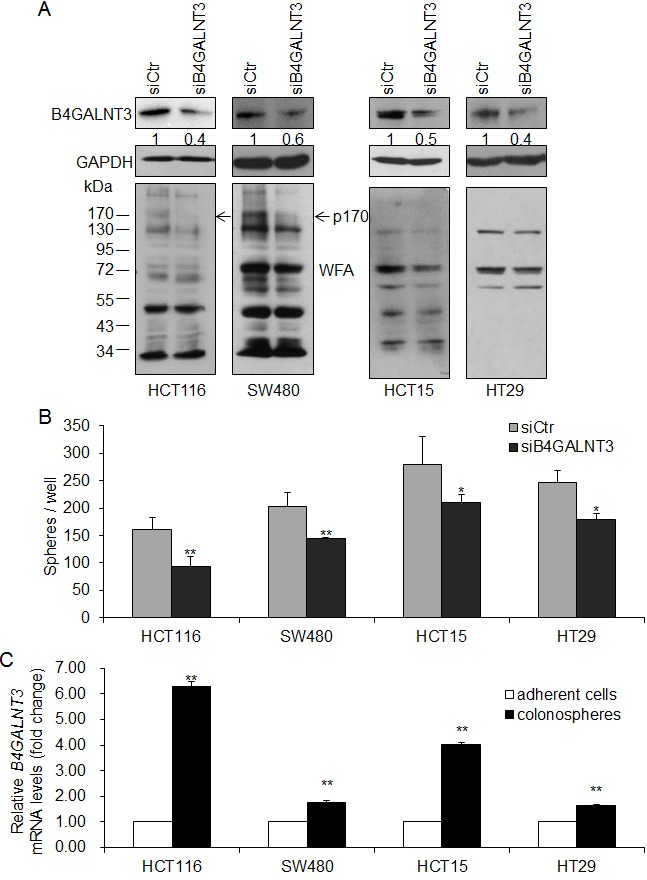
B4GALNT3 knockdown decreases sphere formation in colon cancer cells (A) Transient knockdown of B4GALNT3 in colon cancer cells. Knockdown of B4GALNT3 is analyzed by Western blotting in HCT116, SW480, HCT15, and HT29 cells (upper panel). Relative intensity of B4GALNT3 signals was quantified by ImageJ 1.42q software. Changes in LacdiNAc structure on cellular proteins were confirmed by Western blotting with *Wisteria floribunda* agglutinin (WFA) (lower panel). GAPDH is an internal control. Arrows indicate a 170-kDa protein (p170) with decreased WFA binding in both HCT116 and SW480 cells. (B) Knockdown of B4GALNT3 reduces sphere formation in HCT116, SW480, HCT15 and HT29 cells. Cells (1 × 10^3^ cells/well) knocked down with siCtr (non-targeting control siRNA) or siB4GALNT3 (siRNA against *B4GALNT3*) were cultured in serum-free DMEM/F12 supplemented with B27, 25 ng/mL bFGF, and 20 ng/mL EGF for 7 days. Data from three independent experiments are presented as mean ± standard deviation (SD). *P < 0.05, **P < 0.01 compared with siCtr. (C) The expression of B4GALNT3 increases in colonospheres. Colon cancer cells were cultured in serum-free DMEM/F12 supplemented with B27, 25ng/mL bFGF, and 20 ng/mL EGF for 14 days. The level of B4GALNT3 mRNA expression was analyzed by real time RT-PCR. Data from three independent experiments are presented as mean ± standard deviation (SD). **P < 0.01 compared with adherent cells.

Knockdown of B4GALNT3 expression in HCT116, SW480, HCT15, and HT29 cells were confirmed by Western blotting (Figure 2A, upper penal) and real-time RT-PCR (Figure S1A-D). We found that B4GALNT3 knockdown decreased LacdiNAc expression of several glycoproteins by biotinylated WFA blotting (Figure 2A, lower penal). OCT4 and NANOG are stem cell associated markers and knockdown of B4GALNT3 suppressed its expression in mouse embryonic stem cells [13]. We therefore investigate whether the expression of OCT4 and NANOG alters in B4GALNT3 knockdown cells. We found that the expression of *OCT4* and *NANOG* were decreased in B4GALNT3 knockdown cells at mRNA levels (Figure S1A-D), only the expression of *NANOG* did not significantly change in HT29 cells. Moreover, knockdown of B4GALNT3 suppressed sphere formation in HCT116, SW480, HCT15, and HT29 cells (Figure 2B). Overexpression of B4GALNT3 induced the sphere forming ability of HCT116 and SW480 cells reversely (Figure S2A). Since sphere formation assay is used to enrich the stem-like cells of cancer cells and evaluate their self-renewal capacity [16, 17], we investigate whether the expression of B4GALNT3 alters in colonospheres compared with adherent cells. We found that the level of *B4GALNT3* expression was higher in the colonospheres than that in the adherent cells (Figure 2C). The expression of *OCT4* and *NANOG* were also upregulated in the colonospheres (Figure S3A and S3B), indicating colonospheres are stem-like cells. Our data suggest that B4GALNT3 is able to regulate the stem-like property of colon cancer cells.

### B4GALNT3 modulates EGF-induced but not bFGF-induced sphere formation

**Figure 3:**
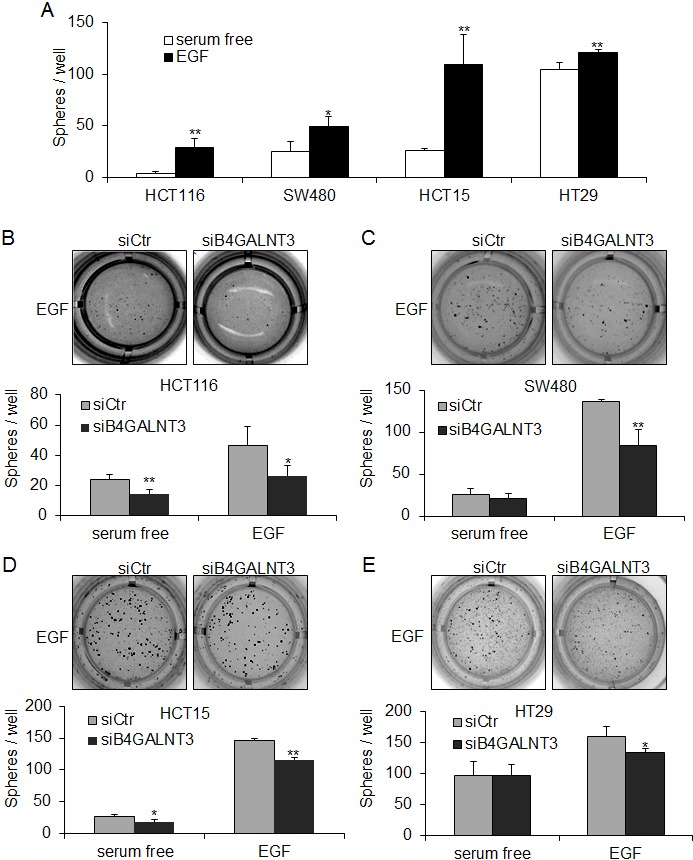
B4GALNT3 regulates EGF-induced sphere formation in colon cancer cells (A) EGF induces sphere formation in HCT116, SW480, HCT15, and HT29 cells. Cells (1 × 10^3^ cells/well) were cultured in serum-free DMEM/F12 containing B27 with or without 20 ng/mL EGF for 7 days. Data from three independent experiments are presented as mean ± standard deviation (SD). *P < 0.05, **P < 0.01 compared with cells cultured in serum free condition. (B-E) Knockdown of B4GALNT3 reduces EGF-induced sphere formation in HCT116, SW480, HCT15, and HT29 cells. Cells (1 × 10^3^ cells/well) knocked down with siCtr (non-targeting control siRNA) or siB4GALNT3 (siRNA against *B4GALNT3*) were cultured in serum-free DMEM/F12 supplemented with B27 and 20 ng/mL EGF for 7 days. The representative images of colonospheres of colon cancer cells are shown (upper) and counted (lower). Data from three independent experiments are presented as mean ± standard deviation (SD). *P < 0.05, **P < 0.01 compared with siCtr.

EGF induced sphere formation in HCT116, SW480, HCT15, and HT29 cells compared with serum free control (Figure 3A). EGFR is a ~170 kDa glycoprotein that regulates stem-like properties in many types of cancers [14, 15]. Notably, a protein with a molecular weight of 170 kDa showed decreased LacdiNAc structure in both HCT116 and SW480 cells (Figure 2A, lower penal). We therefore investigated whether B4GALNT3 could regulate stem-like potential of colon cancer cells via the EGF/EGFR pathway. We found that B4GALNT3 knockdown suppressed EGF-induced sphere formation in HCT116, SW480, HCT15, and HT29 cells (Figure 3B-E). B4GALNT3 knockdown did not alter bFGF-induced sphere formation in colon cancer cells (Figure S4). Conversely, overexpression of B4GALNT3 increased the EGF-induced sphere forming ability of HCT116 and SW480 cells (Figure S2B and S2C), confirming the role of B4GALNT3 in EGF-induced sphere formation. Our results suggest that B4GALNT3 plays a role in regulating cancer stem like cell property via the EGF/EGFR pathway.

### B4GALNT3 knockdown suppresses EGF-induced migration and invasion

**Figure 4:**
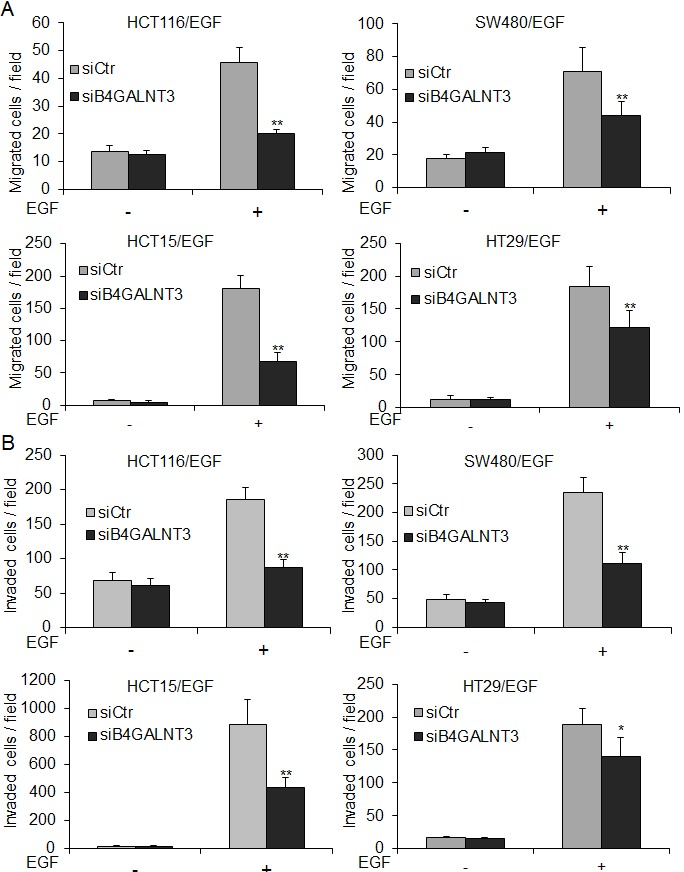
B4GALNT3 knockdown decreases EGF-induced migration and invasion in colon cancer cells (A) Effects of B4GALNT3 on cell migration analyzed by transwell migration assays. HCT116, SW480, HCT15, and HT29 cells in 1% FBS were seeded into the upper chamber and 1% FBS containing with or without 20 ng/mL EGF in the lower chamber was used as chemoattractants. After 48 h, migrated cells were fixed and stained. The migrated cells of siCtr and siB4GALNT3 transfectants were counted. (B) Effects of B4GALNT3 on cell invasion analyzed by matrigel invasion assays. The invaded cells of siCtr and siB4GALNT3 transfectants were counted. The results of three independent experiments are presented as mean ± SD. *P < 0.05, **P < 0.01 compared with siCtr.

We found that B4GALNT3 could regulate EGF-induced sphere formation. Since EGFR also plays an important role in cell survival, migration, and invasion [14, 15], we analyzed whether B4GALNT3 knockdown could inhibit EGF-induced malignant phenotypes. Transwell migration and Matrigel invasion assays indeed showed that B4GALNT3 knockdown significantly decreased EGF-induced migration and invasion in HCT116, SW480, HCT15, and HT29 cells (Figure 4). Since we did not detect significant effect of B4GALNT3 on cell viability by trypan blue exclusion assays in a 72-h period[12]. These results demonstrate that B4GALNT3 is able to regulate colon cancer cell behaviors via the EGF/EGFR pathway.

### B4GALNT3 modifies glycosylation and activity of EGFR in colon cancer cells

**Figure 5:**
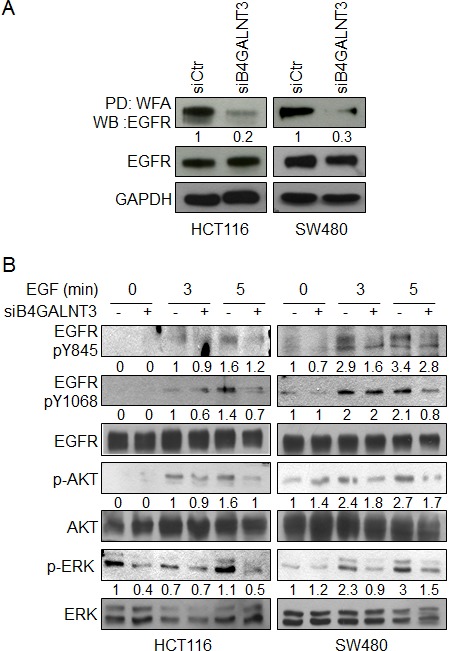
Effects of B4GALNT3 on EGFR glycosylation and signaling (A) B4GALNT3 knockdown suppresses binding of WFA to EGFR in HCT116 and SW480 cells. Equal amounts of cell lysates from siCtr and siB4GALNT3 transfectants were pulled down with WFA lectins and then subjected to Western blotting with anti-EGFR antibody. GAPDH is an internal control. (B) B4GALNT3 knockdown decreases phosphorylation of EGFR in HCT116 and SW480 cells. Cells were serum starved for 8 h and then treated with 5 ng/mL EGF for indicated time points. Phosphorylation of EGFR, AKT and ERK were analyzed by Western blotting. The signals were quantified by ImageJ 1.42q software and normalized to their controls.

Since B4GALNT3 modulated EGF-induced sphere formation and phenotypes, we investigated whether B4GALNT3 could modify EGFR glycosylation and its activity. WFA lectin pull down assay showed that the level of EGFR molecules pulled down in B4GALNT3 knockdown cells was significantly lower than that of the control cells (Figure 5A and Figure S5A), suggesting that the knockdown of B4GALNT3 suppressed the expression of LacdiNAc on EGFRs. In contrast, overexpression of B4GALNT3 increased LacdiNAc on EGFRs (Figure S6). To further investigate the expression of LacdiNAc on *N*- or *O*-glycans of EGFR, PNGase F and benzyl-alpha-GalNAc were used, respectively. Removal of *N*-glycans by PNGaseF almost completely blocked WFA binding to EGFR (Figure S6). However, WFA binding to EGFRs from cells treated with benzyl-alpha-GalNAc was only slightly lower than that of the untreated cells. These findings suggest that B4GALNT3 generates LacdiNAc structure primarily on *N*-glycans of EGFR.

Since the level of EGFR expression was higher in HCT116 and SW480 cells than in HCT15 and HT29 cells (Figure S5B). The effects of B4GALNT3 on EGF-induced phosphorylation of EGFR and its downstream molecules AKT and ERK in HCT116 and SW480 cells were analyzed. Our data showed that B4GALNT3 knockdown suppressed EGF-induced phosphorylation of EGFR at Y845 and Y1068 (Figure 5B). Furthermore, phosphorylation of AKT and ERK was also decreased. These findings suggest that B4GALNT3 knockdown suppressed EGF-induced phosphorylation of EGFR and its downstream signaling molecules AKT and ERK.

### Knockdown of B4GALNT3 promotes EGFR degradation in colon cancer cells

**Figure 6:**
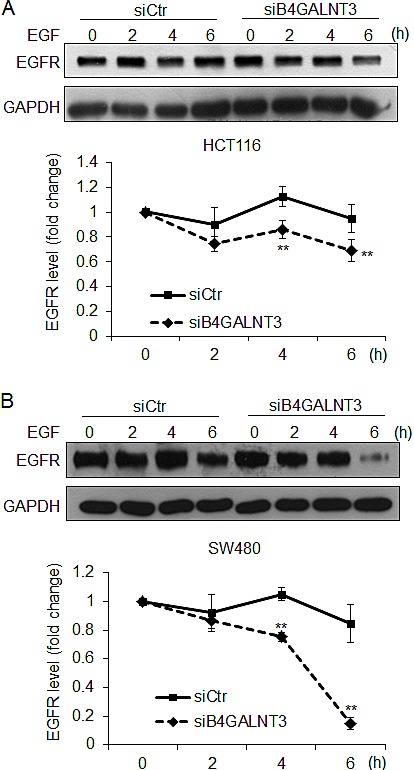
B4GALNT3 knockdown enhances protein degradation of EGFR HCT116 cells (A) and SW480 cells (B) were treated with cycloheximide (20 μg/mL) and EGF (20 ng/mL) for indicated time points. Representative Western blots showed protein levels of EGFR (upper panels) and the signals were quantified by ImageJ 1.42q software (lower panels). EGFR degradation curves in cells transfected with siCtr (solid line) or siB4GALNT3 (dashed line) are shown. Data from three independent experiments are presented as mean ± SD. **P < 0.01 compared with siCtr.

Since *N*-glycosylation has been shown to regulate EGFR dimerization and degradation, we next investigated whether B4GALNT3 could modulate these properties. We found that B4GALNT3 did not significantly affect EGFR dimerization (data not shown). To analyze whether LacdiNAc decoration could affect EGFR stability, we performed protein degradation assays in HCT116 and SW480 cells. Our results showed that B4GALNT3 knockdown promoted EGFR degradation in both HCT116 and SW480 cells (Figure 6). These results suggest that B4GALNT3 knockdown facilitates EGF-induced EGFR degradation in colon cancer cells.

## DISCUSSION

Increasing evidence suggests a rare subpopulation of tumor cells with the stem cell-like characters to initiate xenograft tumor growth [16, 17]. Our previous study showed that overexpression of B4GALNT3 enhances malignant phenotypes of colon cancer cells and increases tumor growth in a mouse xenograft model [12]. Here, we further demonstrated that B4GALNT3 is upregulated in advanced stages of colorectal cancer. This raises the possibility that regulation on B4GALNT3 expression may be crucial for tumor progression of colorectal cancer. We also found that B4GALNT3 plays an important role in regulating the colon cancer stem-like cell property by modulating the LacdiNAc structure on EGFR. It has been shown that EGFR and its ligands are overexpressed in many cancers, such as breast cancer, gastric cancer, ovarian cancer, colorectal cancer, and non-small-cell lung carcinoma [18]. Aberrant activation of EGFR in various tumors regulates cancer stem cell properties [19-21]. In the growth of embryonic stem cells and cancer stem cells, EGF is crucial to maintain the stemness [16, 22]. Moreover, EGFR has been found to regulate the capacity of stemness in cancer cells through AKT and ERK signaling [19, 21]. Consistent with these findings, we showed that B4GALNT3 knockdown suppressed EGF-induced phosphorylation of EGFR, AKT and ERK, and inhibited EGF-stimulated sphere formation. These results suggest that B4GALNT3 is able to regulate stemness of colon cancer cells via the EGFR signaling pathway.

*N*-glycosylation has been reported to regulate several properties of EGFR, including conformation, transportation to cell surfaces, ligand binding, dimerization, endocytosis, and degradation [23, 24]. A previous study showed that B4GALNT3 transfers GalNAc to *N*-glycans on glycoproteins in Chinese hamster ovary (CHO) cells [25]. In the present study, we found for the first time that EGFR carries the LacdiNAc structure. Furthermore, removal of *N*-glycans by PNGaseF almost completely abolished WFA lectin binding to EGFR, indicating that the LacdiNAc structure is mainly decorated on *N*-glycans of EGFR. We therefore suggest that B4GALNT3 changes the LacdiNAc on *N*-glycans of EGFR, modulates its conformation, and thereby alters its kinase activity and degradation.

In addition to *N*-glycans, B4GALNT3 can transfer GalNAc to GlcNAc in core 2 *O*- glycans in CHO cells [26]. We recently showed that EGFR is decorated with mucin-type *O*-glycans, and the *O*-glycans and activity of EGFR can be modified by *N*-acetylgalactosaminyltransferase 2 (GALNT2) in hepatocellular carcinoma [27]. Here we found that benzyl-alpha-GalNAc, an *O*-glycan biosynthesis inhibitor, slightly inhibits WFA binding to EGFR, suggesting the presence of LacdiNAc on *O*-glycans of EGFR. Therefore, it remains possible that changes in EGFR activity and degradation mediated by B4GALNT3 may partially result from modification of *O*-glycans on EGFR. Our result showed that B4GALNT3 knockdown reduced LacdiNAc structure on EGFR in colon cancer cells. However, we did not observe EGFR expression in HCT15 and HT29 cells by WFA lectin blotting. It is possible that a relatively low LacdiNAc structure was expressed on glycoproteins in HCT15 and HT29 cells, hence the protein with a molecular weight of 170 kDa did not found in both total cell lysates.

Carbohydrates on EGFR may modulate EGFR functions via carbohydrate-to-carbohydrate interaction. GM3 is a ganglioside that interacts with EGFR by binding to terminal GlcNAc on EGFRs in A431 cells [28]. This study provides evidence of interaction between GM3 and *N*-glycans inhibits EGF-induced EGFR activity. It is likely that B4GALNT3 knockdown exposes terminal GlcNAc on EGFR, which in turn enhances the interaction between EGFR and GM3 and decreases EGFR activity.

In addition to EGFR, several important glycoproteins, such as MET, insulin-like growth factor-2 receptors (IGF-2R), and vascular endothelial growth factor receptor 2 (VEGFR2), also contain *N*-glycans and play critical roles in the pathogenesis of colorectal cancer [29-31]. Our data showed that several glycoproteins in colon cancer cells can be bound by WFA lectin, and B4GALNT3 modifies the LacdiNAc on these molecules although p170 is the predominant one. Therefore, we still cannot rule out the possibility that, in addition to EGFR, other molecules in colon cancer cells are involved in regulating cancer stem-like cell properties. To unravel the detailed mechanism by which B4GALNT3 modulates cancer stemness, it will be of great interest to further identify the acceptor substrates of B4GALNT3 by glycoproteomics.

The structure of LacdiNAc is frequently found in glycoproteins of tumorous cells, including recombinant EPO from ovarian carcinoma cells [32], ribonuclease 1 from pancreatic adenocarcinoma cells [33], and tissue plasminogen activator from Bowes melanoma cells [34]. The expression of LacdiNAc is also found in glycodelin [35] and is up-regulated in gynecological tumors [36]. However, the effect of LacdiNAc structure on protein functions and cancer phenotypes remains largely unclear. In this study, we first showed that B4GALNT3 modulates the LacdiNAc on EGFR, influences its activity and signaling in colon cancer cells, and in turn alters cancer stemness property. Our findings provide evidence that the LacdiNAc structure on glycoproteins can regulate cancer behaviors via modulating protein functions and signal pathways in the cell.

In this study, we showed that B4GALNT3 regulates cancer cell property via EGFR pathway. Since anti-EGFR antibodies have been shown to improve overall survival in metastatic colorectal cancer [37]. Our findings may suggest an aspect to strengthen anti-EGFR therapy. However, anti-EGFR therapy resistance is associated with KRAS or BRAF mutation [38, 39]. Since colon cancer cell lines used in this study harbored either different KRAS or BRAF mutation (Table 1), our results suggest that B4GALNT3 knockdown suppressed cell malignant phenotype and cancer cell stemness irrelevantly to the status of KRAS and BRAF. Furthermore, B4GALNT3 altered LacdiNAc expression of several glycoproteins, these findings indicated that B4GALNT3 might regulate cancer cell properties through other signaling pathways. It implied that B4GALNT3 could be a novel target for therapy which may be beyond the limitation of KRAS or BRAF mutation.

**Table 1:** KRAS and BRAF status in colon cancer cell lines

	KRAS	BRAF
HCT116	KRAS^G13D^	WT
SW480	KRAS^G12V^	WT
HCT15	KRAS^G13D^	WT
HT29	WT	BRAF^V600E^

In conclusion, we showed that B4GALNT3 is overexpressed in advanced stages colorectal cancer. We have demonstrated for the first time that B4GALNT3 can regulate cancer stem cell properties via modifying EGFR glycosylation and signaling in colon cancer cells. Our findings open novel insights into the significant role of LacdiNAc in cancer stem cells. This study also suggests that targeting B4GALNT3 or LacdiNAc may be a promising strategy to suppress or eliminate cancer stem cells in colorectal cancer.

## MATERIALS AND METHODS

### Patients

From December 2005 to August 2010, a total of 56 colorectal cancer patients were treated at our hospital (National Taiwan University Hospital). The use of human tissues for this study was approved by the National Taiwan University Hospital Ethics Committee (NTUHEC) and written consent was obtained from patients before the collection of samples. For immunohistochemistry, specimens were fixed in 4% (w/v) paraformaldehyde/phosphatebuffered saline (PBS) at 48°C overnight.

### Immunohistochemistry

Human colorectal cancer tissue sections were de-paraffinized in xylene and re-hydrated in a series of graded alcohols. After rinsing twice with PBS, the sections were then quenched the activity of endogenous peroxidase with Ultravision Hydrogen Peroxide Block (Thermo scientific, Barrington, IL) for 10 min and incubated with Ultravision Protein Block (Thermo scientific, Barrington, IL) for 10 min to reduce non-specific binding. Sections were incubated with an rabbit anti-B4GALNT3 polyclonal antibody (Sigma, St. Louis, MO, 1:100) diluted with 1% bovine serum albumin (MDBio, Inc., Taipei, Taiwan)/PBS for 16 h at 4 °C. After rinsing twice with PBS, the sections were processed using the Ultravision Quanta Detection System (Thermo scientific, Barrington, IL). Specific immuno-staining was visualized with DAB Quanto (Thermo scientific, Barrington, IL). All sections were counterstained with hematoxylin for 1 min and mounted with UltraKitt (J.T. Baker, Deventer, Holland). Negative controls were performed by replacing primary antibodies with rabbit IgG (SouthernBiotech, Birmingham, AL) at the same concentration.

### Cell culture and transfection

Colon cancer cell lines HCT116, SW480, HCT15, and HT29 obtained from American Type Culture Collection (Manassas, VA). HCT116, SW480, and HT29 were maintained with Dulbecco's modified Eagle's medium (DMEM) (Thermo scientific, Barrington, IL) containing 10% fetal bovine serum (FBS), 100 IU/mL penicillin and 100 μg/mL streptomycin (Invitrogen, Life Technologies Inc., Grand Island, NY). HCT15 was maintained with RPMI-1640 medium (Thermo scientific, Barrington, IL) containing 10% fetal bovine serum (FBS), 100 IU/mL penicillin and 100 μg/mL streptomycin (Invitrogen, Life Technologies Inc., Grand Island, NY). All cells were incubated in a humidified tissue culture incubator at 37°C and 5% CO_2_ atmosphere. For B4GALNT3 knockdown, duplex siRNA against *B4GALNT3* (siB4GALNT3, 5'-GAG CCA CAU GGA GAC CCA CAA UAA A-3') and non-targeting control siRNA (siCtr) were purchased from Invitrogen. Cells were transfected with siRNAs according to the manufacturer's protocol using Lipofectamine RNAiMAX (Invitrogen, Life Technologies Inc., Grand Island, NY) with a final concentration of 20 nmol/L siRNA for 48 h.

### Real-time RT-PCR

Total cellular RNA was isolated from cells grown to 70% confluence using Trizol reagent (Invitrogen, Life Technologies Inc., Grand Island, NY) and manufacturer protocols as previously described [27]. For cDNA synthesis, 2 μg of total RNA were used as templates in a 20 μl reverse transcription reaction. Quantitative PCR System Mx3000P (Stratagene, La Jolla, CA) was used for real-time PCR reactions. Reactions were performed in a 20-μl volume with 2 μl cDNA, 400 nM each of each sense and anti-sense primers, and 10 μl Brilliant®SYBR®Green QPCR Master Mix (Stratagene, La Jolla, CA). PCRs were incubated for 15 min at 95°C followed by 40 amplification cycles of 30s denaturation at 95°C, 50s annealing at 54°C, and 30s extension at 72°C. Samples were analyzed in triplicate, and product purity was checked by dissociation curves at the end of real-time PCR cycles. Relative quantity of gene expression normalized to GAPDH was analyzed with MxPro Software (Stratagene, La Jolla, CA).

The following primer pairs were used:

*B4GALNT3*, sense (5'-CTACAGCGCATTGTGAACGT-3') and anti-sense (5'-TGGTTCTTCACAGGCACGAC-3');

*OCT4*, sense (5'-GCAGATCAGCCACATCGCCC-3') and anti-sense (5'-GCCCAGAGTGGTGACGGAGA-3');

*NANOG*, sense (5'-GGCCTCAGCACCTACCTACCC-3') and anti-sense (5'-TCCAAGGCAGCCTCCAAGTCA-3');

*GAPDH*, sense (5'-ACAGTCAGCCGCATCTTCTT-3') and anti-sense (5'-GACAAGCTTCCCGTTCTCAG-3').

### Western blot analysis

B4GALNT3 proteins were detected with rabbit anti-B4GALNT3 polyclonal antibody (Sigma, St. Louis, MO). Detection of glycoproteins decorated with LacdiNAc structure was achieved by using biotinylated *Wisteria floribunda* agglutinin (WFA) (Vector laboratories, Burlingame, CA). For EGFR and its downstream signaling analyses, antibodies against total EGFR, EGFR pY 845, EGFR pY1068, p-AKT, AKT, p-ERK1/2, ERK1/2 (Cell Signaling Technology, Danvers, MA), and GAPDH (BD Biosciences, San Jose, CA) were used. Signals on Western blots were quantified by ImageJ 1.42q software (Wayne Rasband, NIH, Bethesda, MA).

### Lectin pull down

Briefly, 500 μg of total cell lysates were incubated with WFA agarose beads (Vector laboratories, Burlingame, CA) overnight at 4°C. The precipitated proteins were then subjected to Western blotting.

### EGFR degradation assays

Cells were seeded on 12-well plates for 24 h, and then serum starved overnight. Cycloheximide (20 μg/mL, Sigma, St. Louis, MO) and 20 ng/mL EGF were added to the cells for the different time points. Cells were lysed in lysis buffer containing 20 mmol/L Tris, pH 8.0, 137 mmol/L NaCl, 1% NP-40, 10%glycerol, 2 mmol/L Na_3_VO_4_, 2 mmol/L β-glycerophosphate, 2 mmol/L PMSF, and 1% protease inhibitor cocktail (Sigma, St. Louis, MO). EGFR protein levels were determined by Western blotting. GAPDH was used as loading control. The 0 h control level was set to 100%, and the detected level of EGFR of each time point was calculated as the percentage of 0 h control.

### Sphere formation assay

Cells were suspended in DMEM/F12 (1:1) supplemented with B27 (Invitrigen, Life Technologies Inc., Grand Island, NY), 25 ng/mL fibroblast growth factor-basic (bFGF, Sigma, St. Louis, MO) and 20 ng/mL epidermal growth factor (EGF, Sigma, St. Louis, MO). The cells were seeded in ultra low attachment 24-well plates (Corning, Tewksbury, MA) at a density of 1,000 cells per well. MTT solution (5 mg/mL, 40 μL, Sigma, St. Louis, MO) were added to each well and pictures were taken after seven days of seeding. The number of spheres with a diameter of >50 μm was quantified by ImageJ 1.42q software (Wayne Rasband, NIH, Bethesda, MA). The results of three independent experiments are presented as means ± standard deviation (SD).

### Formation of colonospheres

Briefly, cells were suspended in sphere medium for 2 weeks. The total RNA was extracted from colonospheres and used for real time RT-PCR analysis.

### Transwell migration assay

Cells (8 × 10^4^, for HCT116, SW480, and HCT15; or 5 × 10^5^, for HT29) in 500 μL DMEM containing 1% FBS were seeded into the upper well of the Boyden chamber with 8-μm pore, 6.5-mm polycarbonate transwell filters (Corning Costar Corp., Cambridge, MA). The lower chambers contained 500 μL DMEM containing 1% FBS and 20 ng/mL EGF (Sigma, St. Louis, MO). After 48 h, cells that had migrated to the lower surface of the membrane were fixed and stained with 0.5% crystal violet (Sigma, St. Louis, MO) and counted under a microscope in six random fields. The results of three independent experiments are presented as means ± SD.

### Matrigel invasion assay

Cell invasion assays were used in BioCoat Matrigel Invasion Chambers (Becton-Dickinson, Bedford, MA) according to the manufacturer's protocol. Briefly, 500 μL containing 1% FBS and 20 ng/mL EGF were added to the lower part of the chamber, and 8 × 10^4^ cells (for HCT116, SW480, and HCT15 cells) or 5 × 10^5^ cells (for HT29 cells) in 500 μL containing 1% FBS were seeded to the upper part. Cells were allowed to invade the matrigel for 48 h. Cells that invaded to the lower surface of the membrane were fixed and stained with 0.5% crystal violet (Sigma, St. Louis, MO) and counted under a microscope in six random fields. The results of three independent experiments were presented as mean ± SD.

### Statistical analyses

Student's *t*-test was used for statistical analyses. Data are presented as mean ± SD. Chi-square tests were used to test associations between B4GALNT3 overexpression and tumor stage. The Kaplan-Meier curve was used for metastasis-free survival analysis, and the log-rank test was used to compare the difference. P < 0.05 was considered statistically significant.

## SUPPLEMENTARY FIGURES


