# Supplementary material for: β1, 4-N-acetylgalactosaminyltransferase III modulates cancer stemness through EGFR signaling pathway in colon cancer cells
Source: Oncotarget. 2014 May 18;5(11):3673–84. doi: 10.18632/oncotarget.1981 (PMC4116512; doi:10.18632/oncotarget.1981)
Supplement: Supplementary file 1 [file oncotarget-05-3673-s001.pdf]

β1, 4-*N*-acetylgalactosaminyltransferase III modulates cancer stemness through EGFR signaling pathway in colon cancer cells

Supplementary Material

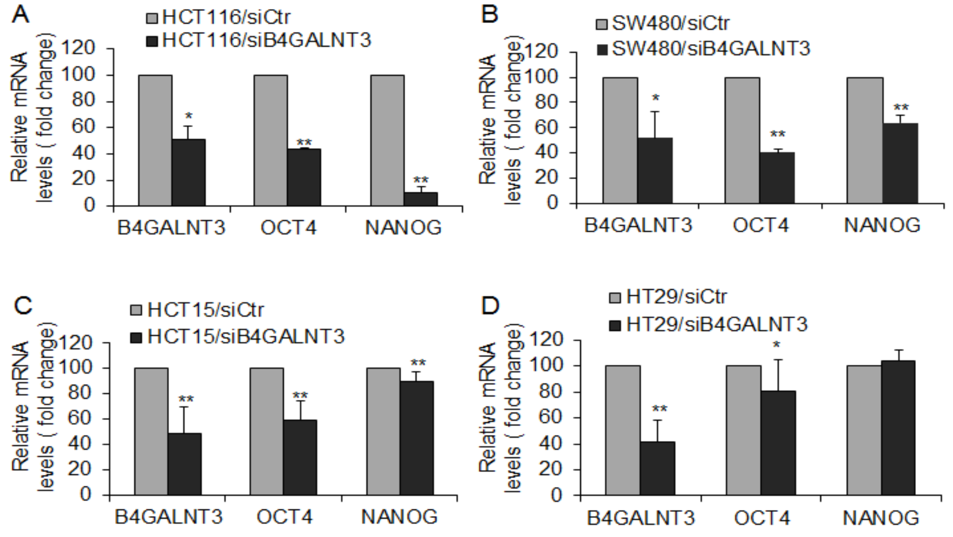

Supplemental Figure S1: Knockdown of B4GALNT3 decreases stem cell markers in colon cancer cells. (A-D) Knockdown of B4GALNT3 reduces OCT4 and NANOG expression in HCT116, SW480, HCT15, and HT29 cells. Real-time RT-PCR analysis of *B4GALNT3*, *OCT4*, and *NANOG* mRNA in colon cancer cells. Data from three independent experiments are presented as mean ± standard deviation (SD). *P < 0.05, **P < 0.01 compared with siCtr.

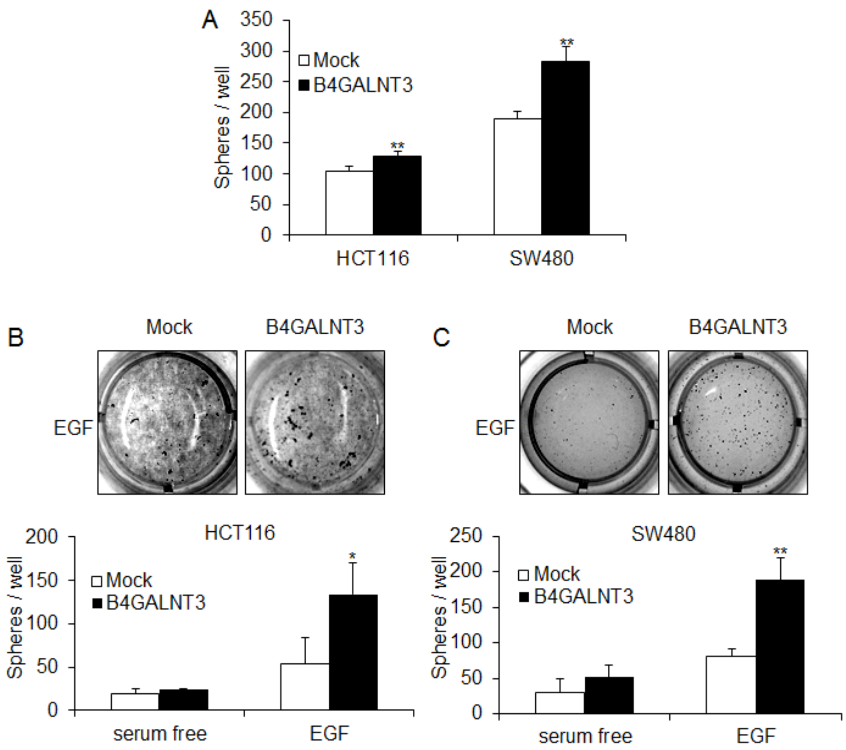

Supplemental Figure S2: Overexpression of B4GALNT3 enhances sphere formation in colon cancer cells. (A) Overexpression of B4GALNT3 increases sphere formation in HCT116 and SW480 cells. Cells (1×10^3cells/well) stably transfected with Mock (pcDNA3.1/myc-His plasmid) or B4GALNT3 (B4GALNT3/pcDNA3.1 plasmid) were cultured in serum-free DMEM/F12 supplemented with B27, 25 ng/mL bFGF, and20 ng/mL EGF for 7 days. Data from three independent experiments are presented as mean ± SD. **P < 0.01 compared with Mock. Overexpression of B4GALNT3 enhances EGF-induced sphere formation in HCT116 (B) and SW480 (C) cells. Cells were cultured in serum-free DMEM/F12 supplemented containing B27 with or without 20 ng/mL EGF for 7 days. The representative images of colonospheres are shown (upper) and counted (lower). Data from three independent experiments are presented as mean ± SD. *P < 0.05, **P < 0.01 compared with Mock.

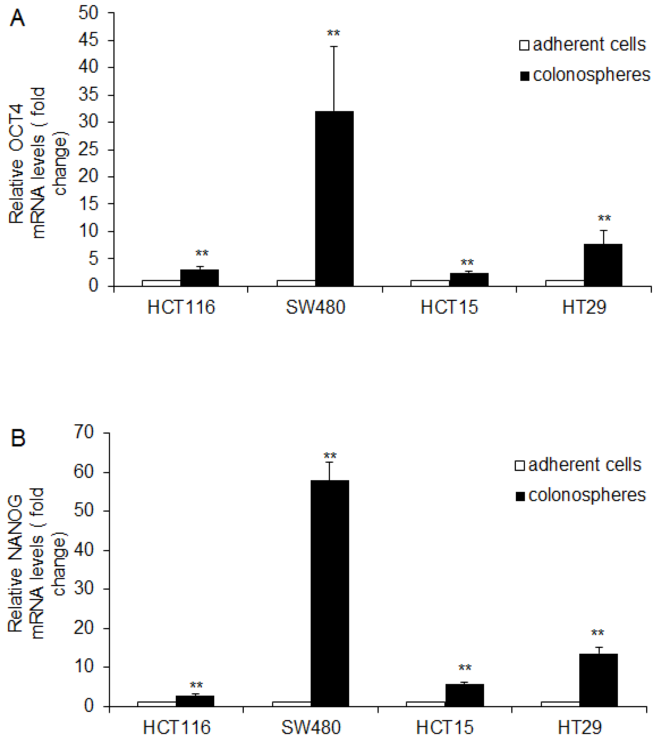

Supplemental Figure S3: Stem cell markers are upregulated in colonospheres. The expression of OCT4 (A) and NANOG (B) are higher in colonospheres than in adherent cells. Colon cancer cells were cultured in serum-free DMEM/F12 supplemented with B27, 25 ng/mL bFGF, and 20 ng/mL EGF for 14 days. The level of *OCT4* and *NANOG* mRNA expression was analyzed by real time RT-PCR. Data from three independent experiments are presented as mean ± standard deviation (SD). **P < 0.01 compared with adherent cells.

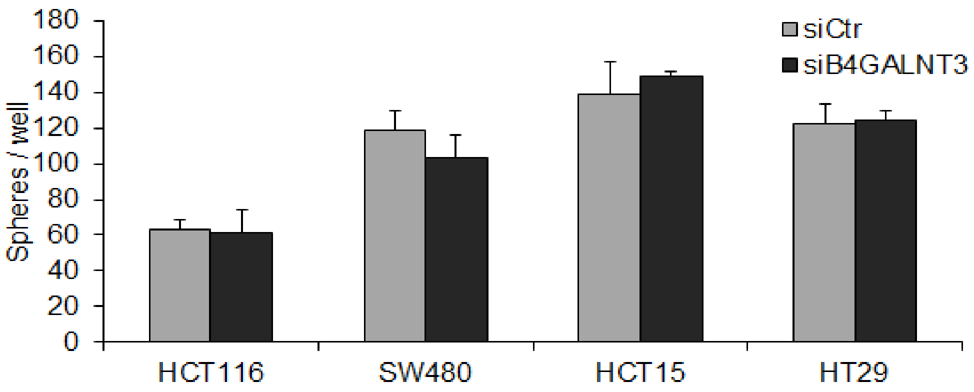

Supplemental Figure S4: **B4GALNT3 does not regulate bFGF-induced sphere formation in colon cancer cells**. Knockdown of B4GALNT3 does not modulate bFGF-induced sphere formation in HCT116, SW480, HCT15, and HT29 cells. Cells (1×10^3 cells/well) knocked down with siCtr (non-targeting control siRNA) or siB4GALNT3 (siRNA against *B4GALNT3*) were cultured in serum-free DMEM/F12 supplemented with B27 and 25 ng/mL bFGF for 7 days. Data from three independent experiments are presented as mean ± standard deviation (SD).

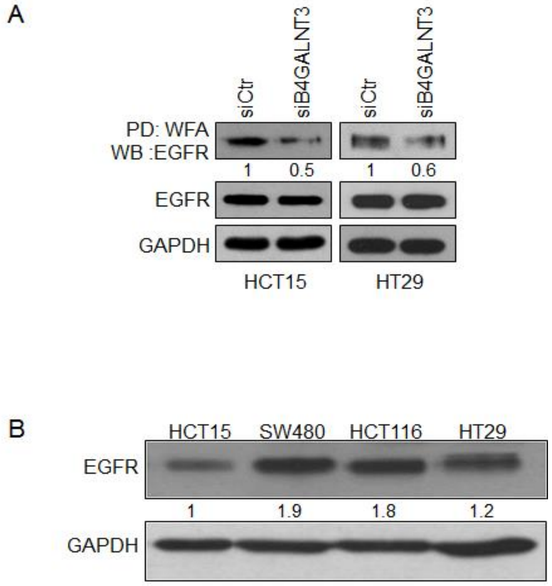

Supplemental Figure S5: **Effects of B4GALNT3 on EGFR glycosylation**. (A) B4GALNT3 knockdown decreases the LacdiNAc decoration on EGFR in HCT15 and HT29 cells. Equal amounts of cell lysates from siCtr and siB4GALNT3 transfectants were pulled down with WFA lectins and then subjected to Western blotting with anti-EGFR antibody. (B) The level of EGFR expression in colon cancer cells. GAPDH is an internal control. Signals on the blots were quantified by ImageJ 1.42q software and normalized to their controls.

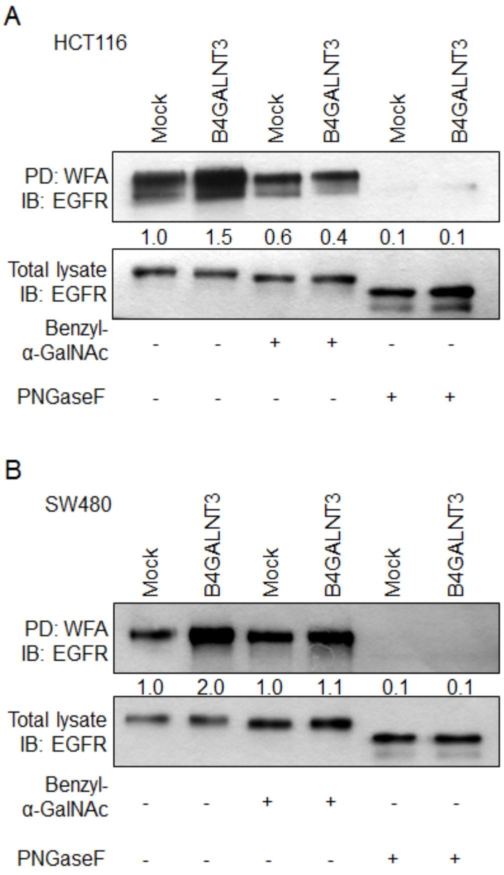

Supplemental Figure S6: B4GALNT3 overexpression increases the LacdiNAc structure mainly on N-glycans of EGFR. B4GALNT3 overexpression enhances binding of WFA to EGFR in HCT116 (A) and SW480 (B) cells. To inhibit mucin-type O-glycan biosynthesis, cells were cultured in DMEM medium containing 10% FBS and 2 mM benzyl-α-GalNAc (Sigma) for 2 days. For removal of N-glycans, cell lysates were treated with N-Glycosidase F (PNGase F, Sigma) at 37°C for 1 hour. Briefly, 500 µg of total cell lysates obtained from benzyl-α-GalNAc-treated cells or incubated with PNGase F were applied to WFA-conjugated agarose beads overnight at 4°C. The pulled down proteins were analyzed by Western blotting with anti-EGFR antibody. Signals on the blots were quantified by ImageJ 1.42q software and normalized to their controls.
